# Intact Neural and Behavioral Processing of Vocal Emotional Expressions in Men with Autism

**DOI:** 10.3390/brainsci16080876

**Published:** 2026-08-18

**Authors:** Silke Vos, Rowena Van den Broeck, Diego Ruiz Callejo, Olivier Collignon, Bart Boets

**Affiliations:** 1 Center for Developmental Psychiatry, Department of Neurosciences, KU Leuven, 3000 Leuven, Belgium; silkevos@hotmail.com (S.V.); rowena.vandenbroeck@kuleuven.be (R.V.d.B.); diego.ruizcallejo@student.kuleuven.be (D.R.C.); 2 Leuven Autism Research (LAuRes), KU Leuven, 3000 Leuven, Belgium; 3 Leuven Brain Institute (LBI), KU Leuven, 3000 Leuven, Belgium; 4 University Psychiatric Center (UPC), KU Leuven, 3000 Leuven, Belgium; 5 Institute of Research in Psychology & Institute of Neuroscience, Université Catholique de Louvain, 1348 Louvain-La-Neuve, Belgium; olivier.collignon@uclouvain.be; 6 School of Health Sciences, HES-SO Valais-Wallis, The Sense Innovation and Research Center, 1007 Lausanne and 1950 Sion, Switzerland

**Keywords:** autism, voice processing, emotion, frequency-tagging EEG, behavioral

## Abstract

**Highlights:**

**What are the main findings?**

- Autistic and non-autistic adult men showed comparable neural and behavioral responses to vocal emotional expressions.
- Frequency-tagging EEG revealed robust automatic discrimination of emotional versus neutral voices in both groups, with strongest responses to fear and anger.

**What are the implications of the main findings?**

- Vocal emotion processing appears largely preserved in autistic adulthood, supporting a developmental-delay rather than persistent-deficit account.
- Auditory frequency-tagging EEG is a sensitive and reliable tool for investigating individual differences in socio-affective processing.

**Abstract:**

Background/Objectives. Human voices convey critical socio-affective information, including emotional states. Although autism has frequently been associated with difficulties in processing vocal emotional cues, findings remain inconsistent, particularly in adults. This study investigated neural and behavioral sensitivity to vocal emotion expressions in autistic adults using an objective auditory frequency-tagging EEG paradigm. Methods. Twenty-five autistic adult men and 25 age- and IQ-matched non-autistic men completed an auditory frequency-tagging EEG task and an auditory and multimodal emotion-recognition assessment. During EEG recording, neutral vocal utterances were presented at 4 Hz, with emotional utterances (fear, anger, happiness, or sadness) inserted every third stimulus, generating an oddball frequency of 1.333 Hz indexing vocal emotion discrimination. Results. No significant group differences were observed in neural or behavioral measures of emotion processing. Robust oddball EEG responses were present in both groups, indicating automatic discrimination of emotional from neutral vocalizations. Fearful and angry vocalizations elicited the strongest neural responses. On the behavioral task, autistic and non-autistic participants showed comparable performance in the auditory modality as well as in the visual and audiovisual modalities, with auditory emotion recognition being the most challenging condition for both groups. Conclusions. These findings provide converging neural and behavioral evidence for intact vocal emotion processing in autistic adult men and are consistent with the view that socio-affective processing differences may attenuate across development. Auditory frequency-tagging EEG shows promise as a sensitive tool for studying individual differences in socio-affective processing.

## 1. Introduction

### 1.1. Vocal Emotion Processing in Autism

Listeners can extract a wealth of information from a human voice, even in the absence of semantic content [1,2]. Beyond cues to identity, gender, and age, voices convey rich socio-affective information about a speaker’s intentions, emotions, and internal states [3,4,5]. The ability to rapidly and accurately process these supralinguistic vocal cues is fundamental for successful social interaction. Difficulties in interpreting such information may therefore contribute to the social communication challenges that characterize autism spectrum disorder (ASD) [6].

ASD is an early-onset neurodevelopmental condition characterized by persistent differences in social communication and interaction, alongside restricted and repetitive patterns of behavior and interests [7]. Difficulties in recognizing and responding to emotional signals are frequently reported and are reflected in the diagnostic criteria relating to nonverbal communication. Consistent with these observations, studies have shown that autistic individuals may experience challenges in identifying emotions from faces, bodies, and voices [8].

Behavioral investigations of vocal emotion recognition in ASD have often reported reduced performance relative to non-autistic individuals [9,10,11,12]. However, findings remain inconsistent with several studies reporting intact or even enhanced performance [13,14]. Such variability likely reflects differences in participant characteristics and methodological approaches [10,14,15,16,17,18,19,20]. Age appears particularly relevant, as vocal emotion recognition continues to develop until approximately 14 to 15 years of age in the general population [21]. Accordingly, larger differences are often observed in autistic children than in autistic adults, suggesting developmental delay rather than a persistent deficit [22]. Gender may also influence outcomes, given the well-established female advantage in emotion recognition [23] and the predominantly male composition of many autism samples [24]. In addition, co-occurring factors such as alexithymia, which affects nearly half of autistic individuals and is associated with depression and anxiety, may contribute to emotion-processing difficulties independently of autism itself [25,26,27,28,29,30,31].

To better understand vocal emotion processing in autism, neurophysiological approaches have increasingly been adopted. Most studies have employed event-related potentials (ERPs) or mismatch negativity (MMN) paradigms [32,33,34,35]. Although these techniques have revealed atypical responses to emotional voices, particularly in children [32], they are constrained by relatively low signal-to-noise ratios and rely on subjective analytical decisions, such as component selection and time-window definition. Consequently, they typically require long recording sessions and may be less suitable for reliable individual-level assessment in clinical populations [36]. These limitations highlight the need for objective and sensitive methods capable of measuring implicit vocal emotion processing efficiently and reliably.

### 1.2. Pinpointing Individual Differences in Socio-Communicative Sensitivity Using Frequency-Tagging EEG

Frequency-tagging EEG combined with fast periodic sensory stimulation provides such an approach. Frequency-tagging capitalizes on the tendency of neural activity to synchronize with periodically presented stimuli, producing responses at predefined stimulation frequencies [37,38]. By presenting different stimulus categories at distinct frequencies, category-selective neural responses can be objectively isolated in the frequency domain.

Building on pioneering work of Barbero et al. (2021) [39], we recently developed and validated an auditory frequency-tagging EEG paradigm that measures neural sensitivity to subtle changes in vocal emotional expressions [40]. In this paradigm, neutral vocal utterances are presented periodically at a base rate of 4 Hz, while emotional utterances are inserted every third stimulus, creating an oddball presentation frequency of 1.333 Hz. Neural activity at the oddball frequency emerges only if the auditory system automatically discriminates emotional from neutral vocalizations, thereby providing a direct index of vocal emotion categorization.

Initial applications of this paradigm demonstrated reduced neural discrimination of vocal emotions in preterm compared with full-term preschool children. Importantly, the amplitude of the emotion-selective EEG response was associated with autism-related traits, as indexed by scores on the Social Responsiveness Scale (SRS-2) [41]. These findings support the sensitivity of the method for detecting individual differences in socio-communicative processing.

The rationale for using frequency-tagging EEG in autism is further supported by evidence from the visual domain. Studies employing fast periodic visual stimulation have consistently identified atypical neural sensitivity to facial identity and facial expressions in autistic children, even when behavioral measures failed to detect group differences [42,43,44,45]. Notably, comparable differences were not observed in autistic adults [46], further supporting the possibility that socio-communicative processing differences may attenuate with development.

Frequency-tagging EEG offers several methodological advantages, including implicit measurement without explicit task demands, objective quantification of responses at predefined frequencies, excellent signal-to-noise ratios, and high test-retest reliability [47]. Furthermore, robust neural responses can be obtained within short recording sessions, making the technique particularly well suited for clinical populations and individual-level assessment.

In the present study, we used this auditory frequency-tagging EEG paradigm to investigate neural sensitivity to vocal emotional expressions in autistic and non-autistic adult men matched for age and IQ. Notably, this is the same adult male cohort that was previously examined by Van der Donck et al. (2023) [46], allowing us to extend earlier findings on neural sensitivity to facial identity and facial expressions to the auditory domain of vocal emotion processing. To complement the neural measures, participants also completed a behavioral emotion recognition task across auditory, visual, and audiovisual modalities. We focused exclusively on male participants because autism is diagnosed more frequently in males than females [24], and because gender has been shown to influence emotion processing and recognition abilities [23]. Restricting the sample to males therefore reduced a potential source of heterogeneity and allowed for a more sensitive examination of autism-related differences in this initial application of the auditory frequency-tagging paradigm.

Based on previous evidence suggesting reduced autism-related differences in adulthood, we expected both groups to show robust neural discrimination of emotional vocalizations and relatively comparable behavioral performance. Nevertheless, given the sensitivity of frequency-tagging EEG to subtle socio-communicative differences, we examined whether group differences could still be detected at the neural level despite comparable behavioral outcomes.

## 2. Materials and Methods

### 2.1. Participants

We recruited 52 male, Dutch-speaking participants (27 ASD and 25 non-autistic (NA)), aged 18–35 years. The sample size was determined based on effect sizes reported in previous auditory frequency-tagging EEG studies [39,40] and group frequency-tagging studies comparing individuals with and without ASD [43,44]. In addition, a sensitivity analysis conducted in G*Power 3.1.7.9 indicated that, with *N* = 52, α = 0.05, and 80% power, the study was sufficiently powered to detect relatively large between-group effects (Cohen’s *d* = 0.79). Consequently, smaller group differences may have remained undetected, and null findings should be interpreted with this consideration in mind. Only male participants were included to minimize potential gender-related variability in vocal emotion processing [23,48,49], and because ASD is diagnosed more frequently in men than in women [24].

Based on self-report, all participants were right-handed, except for one ambidextrous and three left-handed individuals in the ASD group, and one ambidextrous individual in the NA group. All participants underwent pure-tone audiometry, confirming intact hearing abilities (i.e., average PTA hearing loss below 25 dB HL for all participants). Two autistic participants did not complete the second test session, resulting in a final sample of 25 participants per group. As shown in Table 1, groups were successfully matched on age, as well as total, verbal, and performance IQ.

Autistic participants were formally diagnosed by a multidisciplinary team and were referred by the Leuven Autism Expertise Centre (ECA) and the University Psychiatric Centre KU Leuven (UPC Leuven). Four autistic participants had comorbid attention deficit (hyperactivity) disorder (AD(H)D). No other comorbid psychiatric conditions were reported. None of the ASD participants took psychotropic medication. Participants in the comparison group were recruited via social networks and flyers. None reported a current or past psychiatric disorder, nor the use of psychotropic medication. Prior to the experiment, all participants provided written informed consent in accordance with approval from the Medical Ethics Committee UZ/KU Leuven (reference S62969).

All participants completed two test sessions. The first session included IQ assessment, questionnaire completion, hearing evaluation, and administration of a standardized behavioral emotion-processing task. The second session consisted of EEG recording.

### 2.2. Behavioral Assessment

*Intelligence testing*. We administered the subtests block design, similarities, vocabulary and visual puzzles of the WAIS-IV-NL [50] for an estimation of the participants’ verbal, performance and total IQ.

*Quantitative autism questionnaires*. To assess autistic traits, a Dutch version of the Social Responsiveness Scale for adults (SRS-A; [51,52,53]) was administered. This self-report questionnaire measures autistic traits across the subdomains ‘social awareness’, ‘social communication’, ‘social motivation’, and ‘restricted interests and repetitive behavior’. Scores are reported as *T*-scores, with higher scores indicating more pronounced autistic traits. In addition, a Dutch version of the Autism-Spectrum Quotient (AQ; [54,55]) was administered. As with the SRS-A, higher scores reflect a higher level of self-reported autistic traits.

*Alexithymia scale*. A Dutch version of the Toronto Alexithymia Scale (TAS-20; [56]) was administered. This 20-item self-report questionnaire is the most commonly used measure of alexithymia and assesses difficulties in recognizing and expressing emotions [57]. Higher scores indicate more severe alexithymia symptoms.

*Beck Depression Inventory*. Participants completed the Dutch version of the Beck Depression Inventory (BDI-II-NL; [58,59] to assess the severity of depressive symptoms experienced over the past week. Higher scores on this 21-item questionnaire indicate more severe depressive symptoms.

*Pure tone audiometry (PTA)*. Hearing ability was assessed using pure-tone audiometry for both ears (500 Hz, 1000 Hz, and 2000 Hz). None of the participants showed an average pure-tone average (PTA) hearing loss above 25 dB HL.

*Multimodal Emotion Recognition Test (MERT)*. The Multimodal Emotion Recognition Test (MERT; [60]) assesses emotion recognition across auditory, visual and audiovisual modalities, allowing us to determine whether potential group differences were specific to the processing of vocal emotional cues or extended across modalities. Participants identified emotions expressed by actors across ten categories (anger, anxiety, contempt, despair, disgust, elated joy, fear, happiness, irritation, and sadness). Stimuli were presented in four modalities: audio-only recordings, dynamic audiovisual clips, dynamic silent video clips, and static images. A total of 120 items were presented (30 per modality). After each presentation, participants selected the emotion expressed. Performance is reported as the percentage of correctly identified expressions, with higher scores indicating better emotion recognition. For the present study, primary interest focused on performance in conditions involving vocal emotional expressions.

### 2.3. EEG Testing

#### 2.3.1. Stimuli

To create the sequences for the EEG paradigm, we used stimuli from the Emotional Voices and Identity Database (EVID), developed and described by Vos et al. (2023) [40]. This validated database consists of 500 emotionally intoned vocal utterance fragments of 250 ms that can be reliably recognized within this short time window. It includes 10 speakers (5 female), each producing 10 different phonetic utterances, presented with a 10 ms fade-in/out, and expressed with five emotional intonations (neutral, fearful, angry, happy, and sad).

In addition, scrambled versions of the utterances were created following the method described by Dormal et al. (2018) [61]. These sounds preserve the overall frequency content and spectrotemporal structure of the original stimuli but differ in harmonicity. As a result, emotional content is no longer recognizable, while low-level acoustic features are largely maintained.

**Figure 1:**
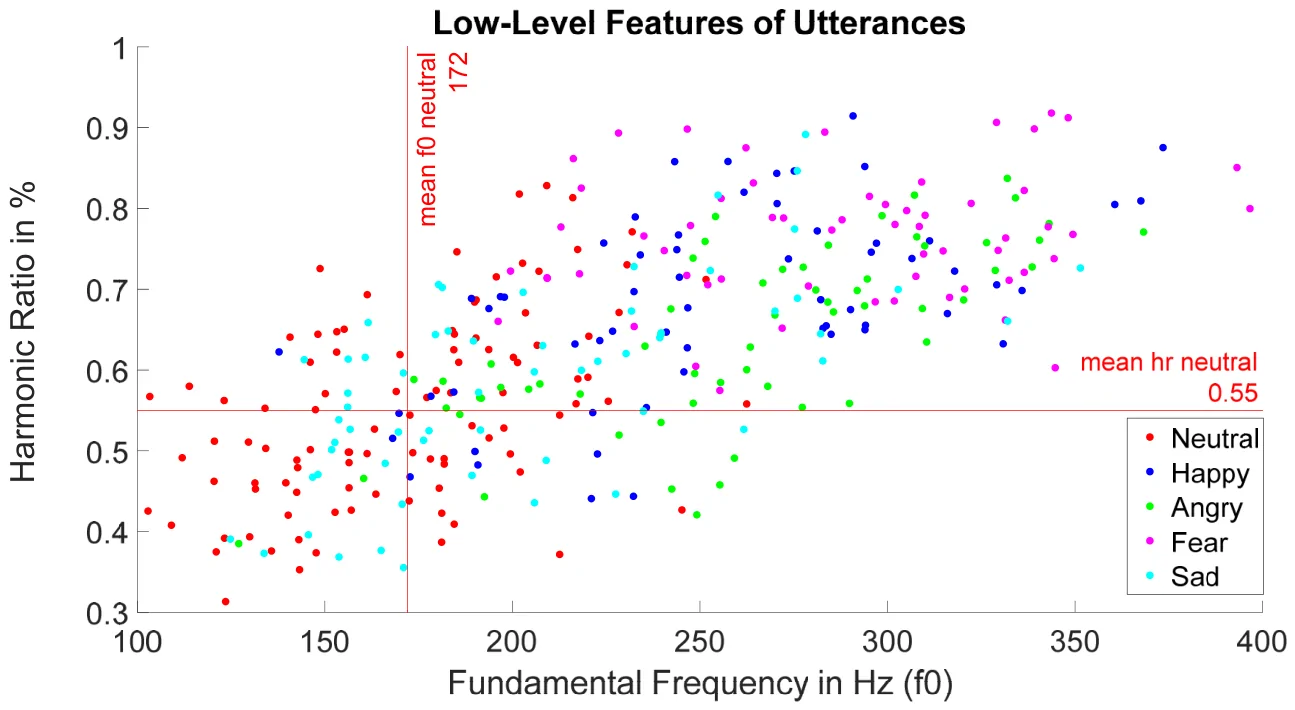
Low-level feature characterization for each of the individual utterances. Each utterance is plotted in terms of pitch (f0) and harmonic ratio (HR in %). The red lines display the value of these features averaged across all neutral utterances.

Emotional vocalizations are typically characterized by features such as fundamental frequency (pitch) and harmonic ratio. To assess the relative contribution of these low-level acoustic properties to emotion discrimination, we calculated the average differences in pitch and harmonic ratio between each emotional category and neutral utterances (see Figure 1). Fundamental frequency (f0) was estimated using the MATLAB function *pitch(audioIn, fs)*, and harmonic ratio (HR) using *harmonicRatio(audioIn, fs)*.

To compute the mean f0 difference, we first calculated the average f0 of all neutral utterances (172 Hz) and subtracted this value from each emotional utterance. For each emotion category, these differences were then averaged across all utterances (*N* = 60). The same procedure was applied for HR.

In terms of f0, fearful utterances differed most from neutral (*MD* = 116, *SD* = 15), followed by angry (*MD* = 89, *SD* = 17), happy (*MD* = 83, *SD* = 17), and sad (*MD* = 33, *SD* = 17). A similar pattern was observed for HR, with fearful utterances showing the largest difference from neutral (*MD* = 0.215, *SD* = 0.025), followed by happy (*MD* = 0.133, *SD* = 0.036), angry (*MD* = 0.090, *SD* = 0.035), and sad utterances (*MD* = 0.034, *SD* = 0.038).

#### 2.3.2. Procedure

**Figure 2:**
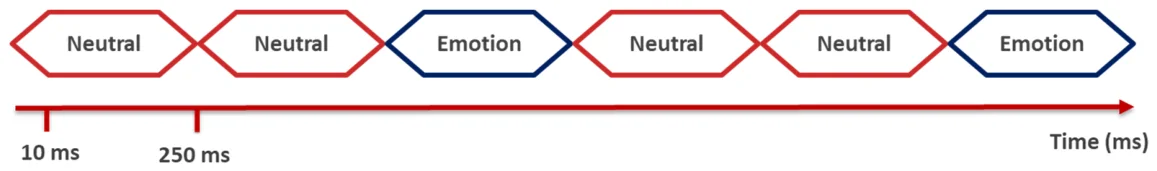
Schematic presentation of the paradigm: Neutral utterances were presented at 4 Hz base rate frequency (in red), with emotional stimuli interleaved every third stimulus (in blue), hence at 1.333 Hz.

Participants listened to 30 sound streams of 64 s, each including a linear fade-in and fade-out of 2 s. For each emotion condition (fearful, angry, happy, sad), six sound streams were presented, comprising three male and three female speakers. For each emotion, we created an oddball paradigm in which emotional utterances were periodically embedded within a stream of neutral utterances. These streams were constructed by concatenating 250 ms neutral utterances, resulting in a base presentation frequency of 4 Hz. Every third stimulus was replaced by an emotional utterance (i.e., NNENNENNE; see Figure 2), yielding an oddball frequency of 1.333 Hz. For each condition, the six speakers with the highest recognition rates for the respective emotion were selected (cf. [40]). Notably, within each sequence, the same speaker was used for both neutral and emotional utterances.

In addition to the four emotional conditions, six scrambled control trials were included using the scrambled stimuli, ensuring that emotional content was no longer recognizable. These trials also comprised three male and three female voices.

To maintain participants’ attention, an orthogonal behavioral task was included that was non-periodic and unrelated to the emotional content of the stimuli. Specifically, participants were instructed to detect brief silent intervals (500 ms) embedded in the sound streams. These occurred randomly four times per sequence (excluding the first and last 5 s and separated by at least 5 s). Participants responded by pressing a button upon detecting a silent interval.

#### 2.3.3. Equipment

We used an ActiveTwo Biosemi system with 64-Ag/AgCl electrodes and two additional electrodes as reference and ground electrodes (Common Mode Sense active electrode and Driven Right Leg passive electrode). Sound sequences were created and presented via a custom-built MATLAB script. Sounds were presented via a calibrated RME Fireface UC with Etymotic Research ER-1 insert earphones to ensure all sounds were presented at an equal intensity of 60 dB SPL. Participants listened to the sound sequences with their eyes closed to avoid eyeblinks, and pressed the button of a Biosemi response switch. The same audio equipment and insert phones were also used for the behavioral testing (PTA hearing levels and MERT).

### 2.4. EEG Analyses

#### 2.4.1. Pre-Processing

The Letswave6 toolbox was used to perform EEG analyses in MATLAB 2019b [62]. As a first step, a fourth-order Butterworth band-pass filter (0.1–100 Hz) was applied to segmented data of 68 s per segment, including 2 s before and 2 s after sound stream onset and offset. The data were then downsampled to 256 Hz and re-referenced to the common average across all electrodes. No eyeblink correction was performed, as participants kept their eyes closed during sound presentation and frequency-tagging EEG is generally robust to eyeblink artifacts.

#### 2.4.2. Frequency-Domain Analyses

Following pre-processing, the data were re-segmented from 2 s after the fade-in to just before the fade-out, resulting in segments of 59.27 s containing an integer number of 1.333 Hz oddball cycles (15,172 time bins). For each participant, the six trials per condition (fear, anger, happy, sad, scrambled) were averaged in the time domain to improve signal-to-noise ratio. For one participant, one trial (female sad condition) was excluded due to a missing trigger, resulting in averaging across five trials for that condition.

The time-averaged data were subsequently transformed into the frequency domain using a Fast Fourier Transform (FFT), yielding an amplitude spectrum with high spectral resolution (0.0167 Hz, i.e., 1/60 s). This approach allows identification of responses at the base frequency (4 Hz) and its harmonics, as well as at the oddball frequency (1.333 Hz) and its harmonics. Importantly, a neural response at the oddball frequency reflects consistent discrimination between base (neutral) and oddball (emotional) stimuli, thereby indexing automatic vocal expression discrimination. For consistency with previous studies, we refer to this as the “expression discrimination response,” although it reflects a distinction between neutral and emotional voices.

To quantify this response, we extracted amplitudes at the oddball frequency and its harmonics. Two complementary measures were computed: (i) signal-to-noise ratio (SNR), defined as the amplitude at a given frequency divided by the average amplitude of the 20 surrounding frequency bins, and (ii) baseline-corrected amplitude, obtained by subtracting this average noise level from the amplitude at the target frequency [63,64]. The surrounding bins consisted of 12 bins on each side, excluding the two adjacent bins and the local minimum and maximum. Individual SNRs and baseline-corrected amplitudes were then averaged per group. SNR spectra were used for visualization, whereas summed baseline-corrected amplitudes across significant harmonics were used for statistical analyses [65].

To determine the harmonics to include, *Z*-scores were computed using the mean and standard deviation of the 20 surrounding bins on grand-averaged FFT data across both groups, all conditions, and all electrodes [64]. Significant responses (Z > 1.64, *p* < 0.05, one-tailed) were observed up to the second harmonic (4 and 8 Hz) for the base response and up to the fourth harmonic for the oddball response. After excluding harmonics overlapping with the base frequency, the oddball response was quantified by summing baseline-corrected amplitudes at 1.333 Hz, 2.666 Hz, and 5.333 Hz.

Regions of interest (ROIs) were defined following the procedure of Vos et al. (2022) [40]. Data from both groups across all conditions were used separately for base and oddball responses. For each electrode, baseline-corrected amplitudes were averaged across participants and conditions and summed across significant harmonics. Electrodes showing amplitudes significantly higher than the mean across all electrodes (Bonferroni-corrected, *p* < 0.001) were selected and grouped into ROIs based on scalp location. In a supplementary analysis, these ROIs were extended by including their contralateral homologues to assess potential hemispheric lateralization effects.

### 2.5. Statistical Analyses

Group differences in questionnaire data were assessed using *t*-tests. MERT data were analyzed using a linear mixed-effects model (LMM), with *Modality* (sound only, dynamic video clips with sound, dynamic video clips without sound, and static pictures) as a fixed within-subject factor, *Group* (ASD vs. NA) as a fixed between-subject factor, and *Participant* included as a random factor with a random intercept. Post hoc *t*-tests were performed and corrected for multiple comparisons using Holm’s correction. Accuracy on the orthogonal task during EEG recording was analyzed using an LMM with *Condition* and *Group* as fixed factors and *Participant* as a random factor.

For the baseline-corrected EEG response amplitudes, two separate LMMs were conducted: one for base-frequency responses and one for oddball-frequency responses. *Condition* (fear, anger, happy, sad, scrambled) and *ROI* were entered as fixed within-subject factors, and *Group* (ASD vs. NA) as a fixed between-subject factor, including their interactions. A random intercept per participant was included to account for repeated measures. Post hoc *t*-tests were corrected using Holm’s procedure. These analyses were first conducted on the core ROIs (comprising all significant electrodes), and subsequently repeated using extended ROIs to examine potential lateralization effects. For oddball responses, additional models were run with and without the scrambled condition to further explore effects specific to emotional conditions.

To examine associations between individual differences in questionnaire measures -autistic traits (AQ, SRS), depression (BDI), and alexithymia (TAS)—and neural vocal emotion discrimination responses, separate LMMs were run in which each questionnaire score was included as a continuous covariate. These analyses were restricted to core ROIs showing significant responses (i.e., LP, MO, and MF).

Finally, significant emotion discrimination responses were assessed at the individual level within the oddball ROIs (e.g., [44,45,63]). For each participant, the raw FFT amplitude spectrum was averaged across electrodes within the ROI and segmented around harmonics of the 1.333 Hz frequency bin, including 20 surrounding bins on each side. Amplitudes across three segments were summed, and the resulting spectrum was converted to a *Z*-score based on the surrounding bins. A response was considered significant if the *Z*-score at 1.333 Hz exceeded 1.65 (*p* < 0.05, one-tailed), indicating signal above noise.

## 3. Results

### 3.1. Behavioral Results

The group results on the questionnaires are presented in Table 2. As expected, and in line with the clinical diagnosis, a highly significant group difference was observed for both SRS and AQ scores. In addition, the autistic group scored significantly higher on the TAS-20 and BDI, indicating more pronounced experienced difficulties in recognizing and expressing emotions, as well as higher levels of depressive symptoms.

Comparison of both groups on the MERT (see Table 2) revealed a significant main effect of *Modality* (*F*(3,144) = 85.81, *p* < 0.001, partial η^2^ = 0.64, 95% CI [0.57, 1]), but no main effect of *Group* (*F*(1,48) = 0.28, *p* = 0.597, partial η^2^ = 0.006, 95% CI [0, 1]) and no *Group* x *Modality* interaction (*F*(3,144) = 0.52, *p* = 0.666, partial η^2^ = 0.01, 95% CI [0, 1]). Post-hoc comparisons showed highest performance for the audio-visual modality (*t*(49) > 3.81, *p* < 0.001 for all contrasts), followed by the visual modality (*t*(49) > 7.38, *p* < 0.001 versus auditory and picture) and the still picture modality (*t*(49) = 3.37, *p* = 0.001 versus auditory). Thus, the auditory-only modality was the most challenging, as reflected in the lowest accuracy scores and consistent with participants’ reports after completing the MERT. As shown in Table 2, both groups performed virtually identically across all modalities.

An LMM of the orthogonal silent gap detection task administered during EEG recording revealed no *Group* differences (*F*(1,46.43) = 0.52, *p* = 0.476; ASD: mean = 93.5%, *SD* = 18.6%; NA: mean = 96.5%, *SD* = 9.1%) and no differences across *Conditions* (*F*(4,616) = 0.28, *p* = 0.892; Angry: mean = 95.2%, *SD* = 12.7%; Fear: mean= 94.5, *SD* = 15%; Happy: mean = 95.1, *SD* = 14.9%; Sad: mean = 94.6%, *SD* = 13.1%; Scrambled: mean = 95.3%, *SD* = 15.8%). These findings indicate comparable attention levels across groups and conditions.

### 3.2. Vocal Expression EEG Results

#### 3.2.1. Region of Interest Delineation

**Figure 3:**
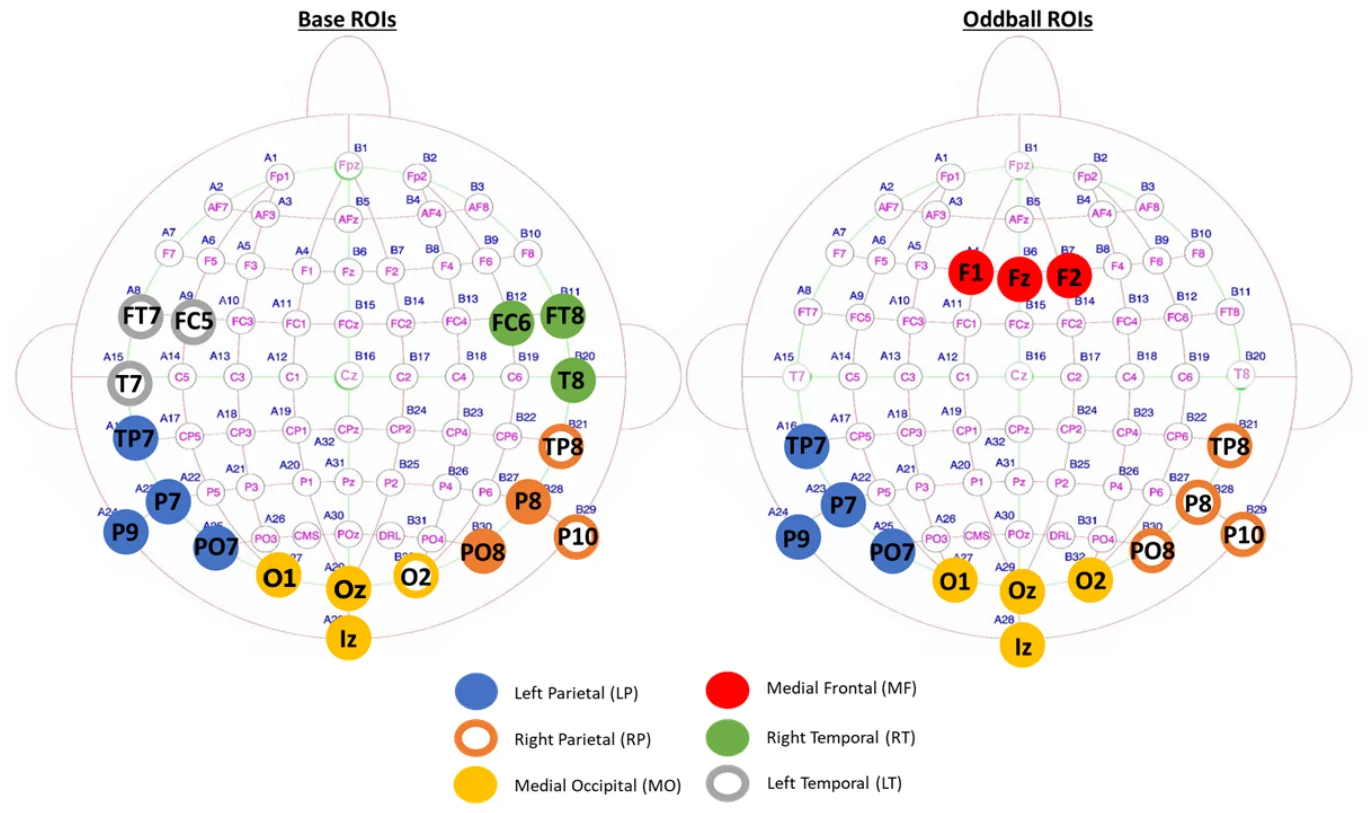
ROI delineation for base and oddball responses separately. Significant electrodes and the core ROIs are depicted in full circles, the extended ROIs are depicted with empty circles. Note that O2 did not reach significance for the base-frequency response but was included in the medial occipital ROI to maintain consistency and limit the total number of ROIs. Figure 3 illustrates the ROI configurations for base and oddball responses, including the extended ROIs.

The objective procedure for defining the regions of interest yielded 12 significant electrodes for the base-frequency response (O1, Oz, Iz, P7, P9, PO7, TP7, P10, TP8, FC6, FT8, T8) and 11 significant electrodes for the oddball-frequency response (O1, O2, Oz, Iz, P7, P9, PO7, TP7, F1, F2, Fz; see Figure 3). Given the substantial overlap between electrodes contributing to the base and oddball responses, these electrodes were grouped into four core ROIs based on their scalp location.

Specifically, for the base responses, three core ROIs were defined: left parietal (LP: P7, P9, PO7, TP7), medial occipital (MO: O1, O2, Iz, Oz), and right temporal (RT: FC6, FT8, T8). For the oddball responses, three core ROIs were delineated: left parietal, medial occipital, and medial frontal (MF: Fz, F1, F2).

To investigate potential hemispheric lateralization effects and to ensure inclusion of all significant electrodes, supplementary analyses were conducted using extended ROIs. These included the contralateral homologue regions, namely right parietal (RP: P8, P10, PO8, TP8) and left temporal (LT: FC5, FT7, T7).

#### 3.2.2. Similar Base Rate Responses in ASD and NA Groups

**Figure 4:**
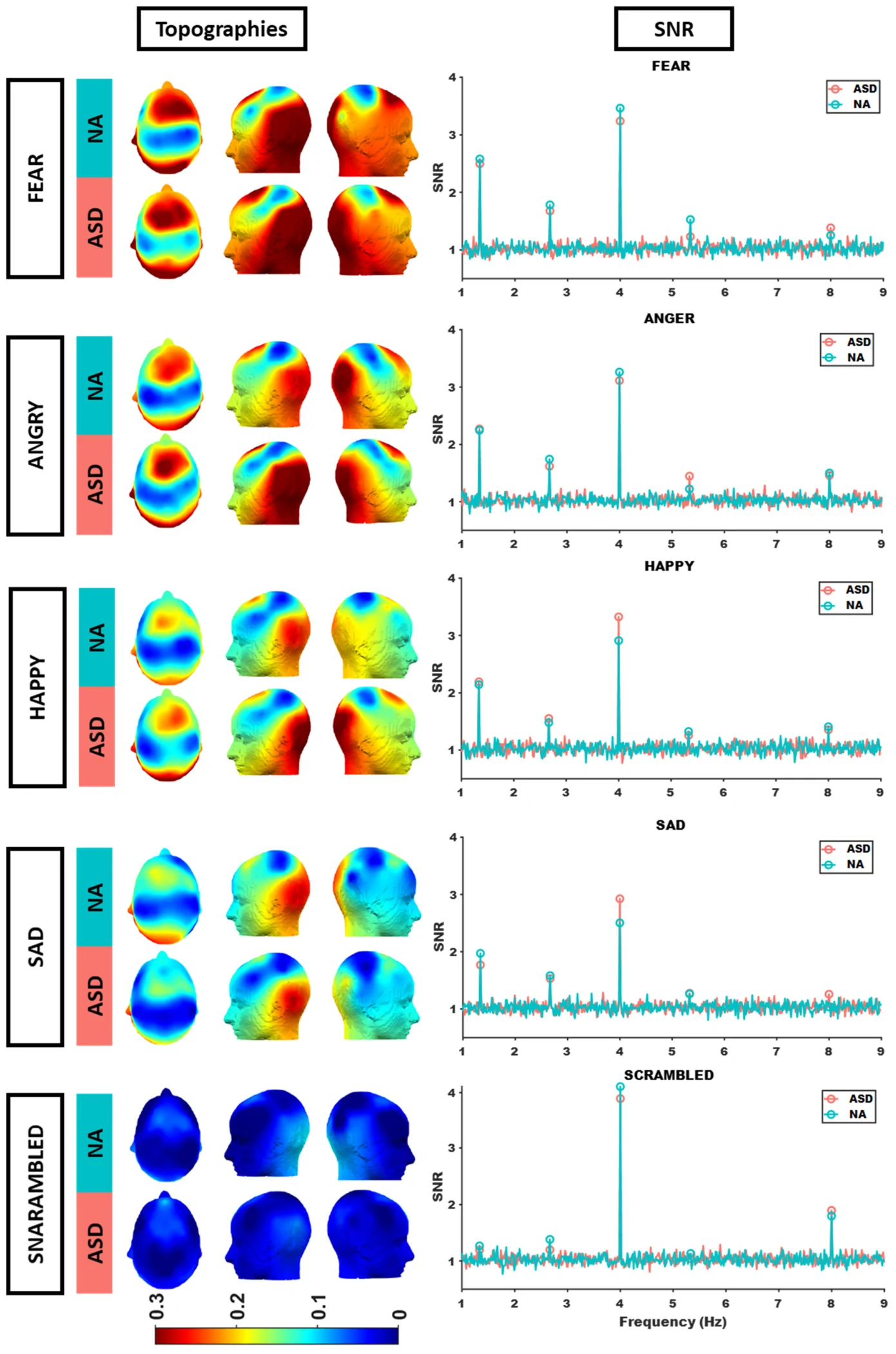
Oddball responses indexing automatic neural discrimination of vocal emotional expressions. **Left**: Scalp topographies showing the spatial distribution of summed baseline-subtracted amplitudes at the oddball frequency and its significant harmonics (1.333, 2.666, and 5.333 Hz) for each condition. Warmer colors indicate stronger neural discrimination responses, whereas cooler colors indicate weaker responses. Topographical maps are displayed separately for the non-autistic (NA; upper row) and ASD (lower row) groups and illustrate the consistent distribution of emotion-discrimination responses across the scalp. Oddball activity was predominantly centered over the left parietal (LP), medial occipital (MO), and medial frontal (MF) regions that were selected as regions of interest (ROIs) for the statistical analyses. **Right**: Signal-to-noise ratio (SNR) spectra for each condition, averaged across the oddball-specific ROIs (MO, LP, and MF). Blue lines represent the mean response of the NA group and red lines represent the mean response of the ASD group. Responses at 1.333, 2.666, and 5.333 Hz oddball frequencies reflect neural discrimination of emotional relative to neutral vocalizations.

General auditory base rate responses were clearly observed in both groups, as reflected by peaks in the SNR spectrum at 4 Hz and 8 Hz (see Figure 4; see Supplementary Figure S1 for topographies per group and condition). A repeated-measures LMM on the base rate responses revealed significant main effects of *Condition* (*F*(4,672) = 14.27, *p* < 0.001, partial η^2^ = 0.08, 95% CI [0.05, 1]), ROI (*F*(2,672) = 10.00, *p* < 0.001, partial η^2^ = 0.03, 95% CI [0.01, 1]), as well as a significant *Condition* × *ROI* interaction (*F*(8,672) = 10.19, *p* < 0.001, partial η^2^ = 0.11, 95% CI [0.07, 1]). No main effect of *Group* (*F*(1,48) = 0.71, *p* = 0.405, partial η^2^ = 0.01, 95% CI [0, 1]) was found, nor were there any significant two- or three-way interactions involving *Group* (*p* > 0.566).

Post-hoc comparisons for the condition effect indicated that the Scrambled condition elicited higher responses than Sad, Happy and Fear (*t*(149) > 2.93, *p* < 0.023 for all contrasts), while the Sad condition yielded the lowest responses (significantly lower than all other conditions, *t*(149) > 5.64, *p* < 0.001 for all contrasts). The ROI effect further showed that base-rate responses were most pronounced in the left parietal ROI (LP) across all conditions except Sad (see Supplementary Figure S2 for spatial distribution across conditions).

To further examine potential group differences in the lateralization of base-rate vocal processing, the analysis was replicated including the extended ROIs (cf. empty circles in Figure 3). Again, no main effect of *Group* (*F*(1,1152) = 0.54, *p* = 0.468, partial η^2^ = 0.01, 95% CI [0, 1]) and no interactions involving *Group* (all *p* > 0.310) were observed. As expected, the effect of ROI became more pronounced and showed that all core ROIs (LP, MO and RT) elicited significantly stronger responses than the extended ROIs (RP and LT) (*F*(4,1152) = 46.28, *p* < 0.001, partial η^2^ = 0.14, 95% CI [0.11, 1]).

#### 3.2.3. Similar Vocal Expression Discrimination Responses in ASD and NA Groups

Neural sensitivity to changes in vocal expression was investigated using responses tagged to the oddball rate. The SNR spectra clearly show vocal expression discrimination responses in both groups, with prominent peaks at the oddball frequency of 1.333 Hz and its harmonics (Figure 4). For the scrambled condition, these peaks were markedly smaller, though still significant at the oddball frequency.

An LMM on the oddball-rate responses revealed a significant main effect of *Condition* (*F*(4,672) = 75.68, *p* < 0.001, partial η^2^ = 0.31, 95% CI [0.26, 1]) and *ROI* (*F*(2,672) = 5.88, *p* = 0.003, partial η^2^ = 0.02, 95% CI [0, 1], but no significant *Condition* × *ROI* interaction (*F*(8,672) = 0.79, *p* = 0.615, partial η^2^ = 0.009, 95% CI [0, 1]). Crucially, no main effect of *Group* was observed (*F*(1,48) = 0.26, *p* = 0.614, partial η^2^ = 0.005, 95% CI [0, 1]), nor were there significant *Group* × *ROI* or *Group* × *ROI* × *Condition* interactions (both *p* > 0.14), indicating that ROI effects were comparable across ASD and NA groups. A small but significant *Group* × *Condition* interaction was observed (*F*(4,672) = 2.43, *p* = 0.047, partial η^2^ = 0.01, 95% CI [0, 1]).

Post hoc paired comparisons showed significantly lower responses for the Scrambled condition compared to all emotional conditions (*t*(149) > 8.94, *p* < 0.001). Fear and anger elicited significantly higher responses than the other emotional conditions (t(149) > 2.63, *p* < 0.013), with fear also yielding higher responses than anger (*t*(149) = 3.05, *p* = 0.008). Additionally, happy expressions elicited higher responses than sad expressions (*t*(149) = 2.76, *p* = 0.013).

With respect to *ROI*, post hoc testing indicated stronger responses in the medial occipital and left parietal regions compared to the medial frontal region (*t*(249) > 4.24, *p* < 0.001). Although the *Group* × *Condition* interaction reached significance, no direct group differences were observed within any of the individual conditions (*t*(74) < 1.84, *p* > 0.070). Within-group comparisons showed that, in the NA group, anger elicited smaller responses than fear (t(74) = 3.15, *p* = 0.010), whereas no such difference was found in the ASD group (*t*(74) = 1.29, *p* = 0.200).

**Figure 5:**
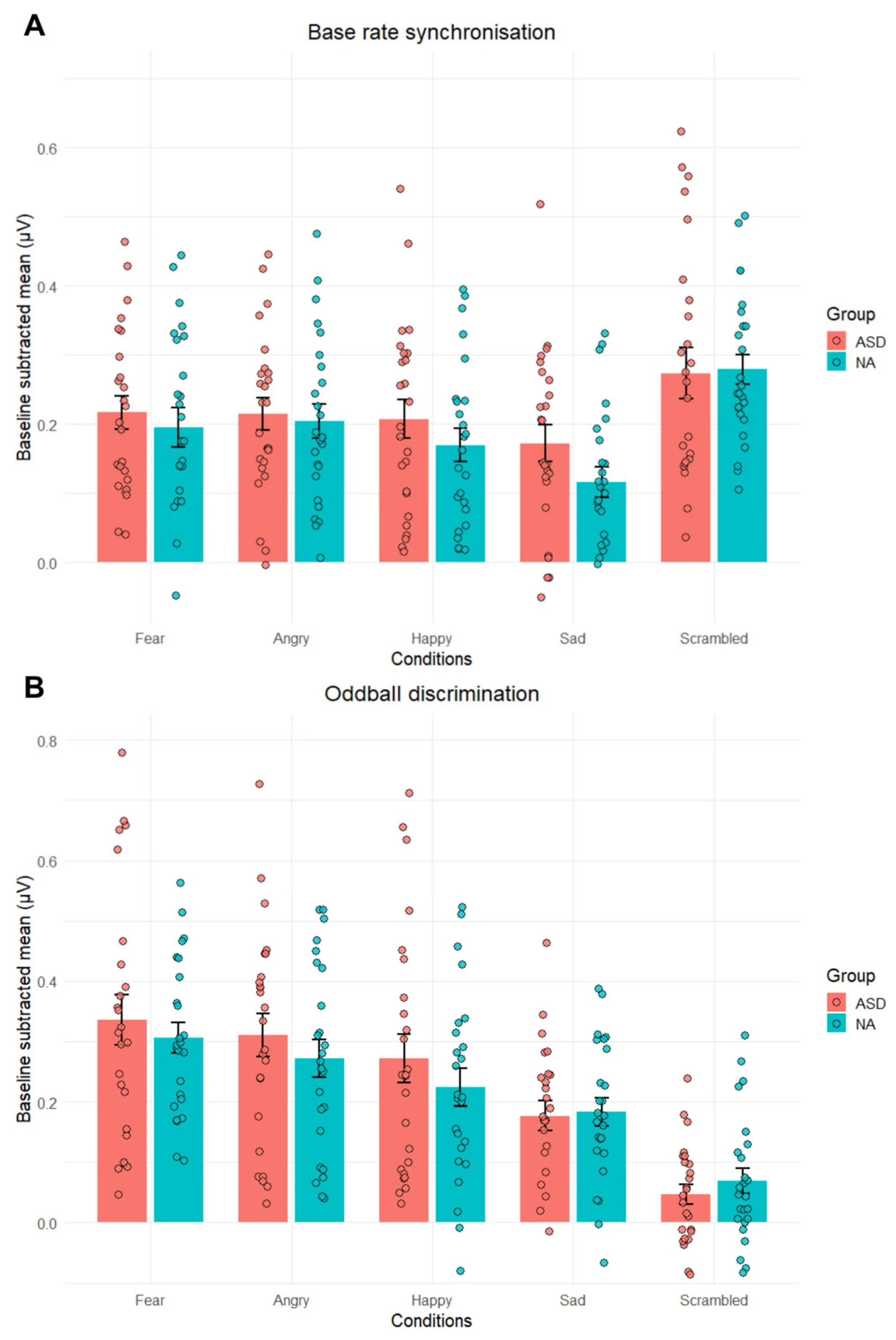
(**A**) Base-rate responses: Mean summed baseline-subtracted amplitudes at the base frequency for all conditions and both groups, averaged over the core base ROIs. (**B**) Oddball discrimination responses: Mean summed baseline-subtracted amplitudes at the oddball frequency for all conditions and both groups, averaged over the core oddball ROIs. Error bars represent the standard error of the mean. Individual participant data points are superimposed on the bar graphs.

Importantly, repeating the analysis on the emotional conditions only (excluding the scrambled condition) did not reveal any significant main or interaction effects involving *Group* (all *p* > 0.082). Figure 5 presents the condition-specific base-rate (top) and oddball-rate (bottom) responses for both groups.

To further examine potential group differences in neural lateralization of vocal emotion discrimination, the analysis was repeated including the extended ROIs (cf. empty circles in Figure 3). Again, no main effect of *Group* (*F*(1,912) = 0.40, *p* = 0.531, partial η^2^ = 0.008, 95% CI [0, 1]) and no interactions involving *Group* (all *p* > 0.138) were observed. As expected, the *ROI* effect became more pronounced, showing that all core ROIs (LP, MO, MF) elicited stronger responses than the extended ROI (RP) (*F*(3,912) = 12.24, *p* < 0.001, partial η^2^ = 0.04, 95% CI [0.02, 1]).

#### 3.2.4. No Significant Association Between Quantitative Autism Characteristics, Alexithymia and Depression Symptoms, and Neural Vocal Emotion Discrimination Responses

To explore potential dimensional associations between individual differences in autism characteristics, alexithymia and depressive symptoms on the one hand, and neural sensitivity to subtle vocal emotional cues on the other, we ran a series of similar LMM analyses in which each questionnaire variable was entered separately as a continuous covariate (excluding the scrambled condition). None of these covariates significantly predicted the amplitude of the neural emotion discrimination response: SRS: *F*(1,47) = 0.13, *p* = 0.723, partial η^2^ = 0.003, 95% CI [0, 1]); AQ: *F*(1,47) = 0.72, *p* = 0.400, partial η^2^ = 0.02, 95% CI [0, 1]); TAS-20: *F*(1,47) = 0.32, *p* = 0.576, partial η^2^ = 0.007, 95% CI [0, 1]); BDI: *F*(1,47) = 0.51, *p* = 0.481, partial η^2^ = 0.01, 95% CI [0, 1]).

#### 3.2.5. Significant Oddball Responses at the Individual Level

It is also important to assess the extent to which reliable emotion discrimination responses can be detected at the individual subject level. Analysis of individual data showed that 48 out of 50 participants (all but two autistic men) exhibited a significant discrimination response in the most robust condition (fear; *Z* > 1.64, *p* < 0.05). The least consistent responses were observed for the scrambled condition (14/50 participants). See Table 3 for results across all conditions.

## 4. Discussion

The present study used auditory frequency-tagging EEG to investigate neural sensitivity to vocal emotional expressions in autistic and non-autistic adult men. By measuring both general voice processing (base-rate responses) and automatic discrimination of emotional versus neutral vocalizations (oddball responses), we found remarkably similar neural response profiles across groups. Both groups exhibited robust base- and oddball-frequency responses across emotional conditions, showed similarly reduced responses to acoustically matched scrambled stimuli, and performed comparably on both an auditory emotion-recognition task and a broader multimodal emotion-recognition assessment. Together, these findings provide converging neural and behavioral evidence for intact vocal emotion processing in autistic adult men.

### 4.1. Intact Vocal Emotion Processing in Autistic Adult Men

Robust base- and oddball-frequency EEG responses were observed across all conditions in both groups. These findings indicate that autistic and non-autistic adults successfully synchronized their neural activity to the temporal structure of vocal stimuli and automatically discriminated subtle emotional vocal cues embedded within rapidly presented streams of neutral utterances. Importantly, no statistically significant group differences were observed for either general voice processing or vocal emotion discrimination.

While the present study was not powered to detect very small group differences, the absence of group effects is unlikely to be attributable to insufficient statistical sensitivity. Notably, the ASD group did not show a trend toward reduced neural responses. If anything, oddball response amplitudes were generally larger in the ASD group than in the non-autistic group, arguing against the presence of even subtle impairments in neural vocal emotion processing.

The neural findings are particularly consistent with performance on the auditory-only condition of the MERT. Autistic and non-autistic participants obtained virtually identical scores when recognizing emotions from voices alone, providing converging behavioral evidence for intact vocal emotion processing in adulthood. Although no group differences were observed in the visual and audiovisual conditions either, these modalities were not the primary target of the present study and therefore mainly serve to demonstrate that the absence of group differences extended beyond the auditory domain. Importantly, both groups showed their lowest performance in the auditory-only condition and highest performance for audiovisual stimuli, confirming that emotion recognition based solely on vocal cues is inherently more challenging than multimodal emotion recognition. Taken together, the convergence between auditory EEG responses and auditory behavioral performance suggests that both early, implicit perceptual encoding and higher-level processes involved in explicitly recognizing and labeling vocal emotions are largely intact in autistic adult men.

When comparing oddball responses across emotional conditions, the strongest responses were observed for fearful and angry vocalizations. This pattern is consistent with previous evidence suggesting preferential processing of threat-related emotions because of their evolutionary significance and increased perceptual salience [44,66,67]. Interestingly, a comparable hierarchy has been reported in visual frequency-tagging studies, where fearful and angry facial expressions evoked stronger neural discrimination responses than happy or sad expressions [44]. The present findings therefore suggest that autistic and non-autistic adults display a similar threat-detection advantage during vocal emotion processing [68,69].

At the same time, acoustic analyses indicated that fearful and angry utterances also differed most strongly from neutral utterances in terms of low-level acoustic features such as pitch and harmonic ratio. Consequently, it remains difficult to fully disentangle the relative contributions of acoustic distinctiveness and higher-level emotional categorization to the observed response pattern. Nevertheless, converging evidence from frequency-tagging studies in the visual domain suggests that threat-related advantages are unlikely to be explained solely by low-level stimulus characteristics [44].

### 4.2. Neural Correlates of Vocal Emotion Discrimination

Although neuroanatomical interpretations of scalp EEG topographies should be made cautiously in the absence of formal source localization, the observed response distributions are broadly consistent with the auditory-social processing network described in previous literature. Base-rate responses, reflecting general voice processing, were centered around left parietal, medial occipital, and right temporal scalp regions. This pattern aligns with evidence that human voices are rapidly processed through bilateral superior temporal regions, including the temporal voice areas located along the superior temporal sulcus and gyrus [39,70,71].

The additional medial frontal activity observed for oddball responses, indexing discrimination between emotional and neutral vocalizations, is consistent with neuroimaging evidence showing that emotional voice processing recruits a broader socio-affective network extending beyond auditory cortex to include medial prefrontal regions, superior temporal cortex, and limbic structures involved in the evaluation of socially relevant affective signals [3,4,72,73,74,75,76,77]. Taken together, the present topographies are compatible with hierarchical models proposing that temporal voice-sensitive regions support the analysis and categorization of vocal signals, whereas frontal regions contribute to the extraction and evaluation of their socio-emotional significance.

Importantly, the absence of group differences across both temporal and frontal response patterns suggests that the large-scale auditory-social network supporting the extraction and evaluation of vocal affective information is functionally preserved in autistic adult men.

### 4.3. Comparable Processing of Low-Level Acoustic Information

Consistent with previous validation work [40], oddball responses were substantially reduced for the scrambled control condition compared with all emotional conditions. Although the scrambled stimuli preserved aspects of the low-level spectrotemporal acoustic structure, they were no longer recognizable as voices and no longer conveyed identifiable emotional content. The persistence of a small but significant oddball response therefore suggests that periodic low-level acoustic differences can contribute modestly to frequency-tagged EEG responses, whereas robust responses require higher-level categorization of emotional vocal content.

Importantly, the similarly reduced scrambled responses observed in autistic and non-autistic participants do not support theories proposing superior low-level auditory processing in autism, such as enhanced pitch perception [78,79,80,81]. Nor do they support the suggestion that autistic individuals primarily rely on low-level acoustic cues as a compensatory strategy when processing emotional speech [82]. If such a strategy were driving performance, one might have expected increased sensitivity to the scrambled stimuli in the ASD group. Instead, both groups showed nearly identical reductions in neural response magnitude.

Furthermore, successful performance in the current paradigm requires generalization across a broad range of utterances produced by different speakers. Unlike previous reports suggesting atypical generalization of auditory information in autism [83], our findings indicate that autistic participants readily generalized across acoustically variable exemplars within emotional categories. However, this ability alone was insufficient to generate strong oddball responses when emotional meaning was removed from the stimuli.

### 4.4. Relating Findings to the Broader Autism Literature

The literature on vocal emotion processing in autism remains characterized by considerable heterogeneity and inconsistent findings [17]. Against this background, the absence of group differences in the present study contrasts with reports of impaired vocal emotion recognition in autism [9,33], particularly in children [22,32,34,35], and in individuals with elevated alexithymia [27].

As emphasized in the Introduction, autism is a developmental condition, and age may play a critical role in explaining inconsistencies across studies. Group differences commonly reported during childhood may diminish or disappear over the course of development. This interpretation is supported by visual frequency-tagging studies showing reduced facial identity and facial expression discrimination in autistic children [42,43,44,45], but not in autistic adults from the same research cohort as investigated here [46]. The current findings extend this pattern to vocal emotion processing and support the hypothesis of a developmental delay rather than a persistent impairment in socio-communicative functioning.

Importantly, comparable developmental patterns have now been observed across both visual and auditory domains, suggesting that maturation of socio-communicative mechanisms may continue well into adulthood for at least a subset of autistic individuals. This interpretation is further supported by recent work in prematurely born preschool children, an at-risk population characterized by subtle socio-communicative vulnerabilities. Using the same auditory frequency-tagging paradigm, Van den Broeck et al. (2026) reported reduced neural discrimination of vocal emotions and significant associations with parent-reported autism characteristics [41]. Together, these findings point toward the possibility that differences in vocal emotion processing are more pronounced earlier in development and may attenuate across maturation.

Another explanation frequently invoked in the autism literature is that emotion-processing difficulties may be driven by co-occurring characteristics such as alexithymia and depression rather than by autism itself [25,26,28,30,31]. Consistent with this account, the autistic group reported significantly higher levels of alexithymia and depressive symptoms than the non-autistic group. However, exploratory dimensional analyses revealed no significant associations between neural vocal emotion discrimination responses and self-reported alexithymia (TAS-20), depressive symptoms (BDI), or autistic traits (AQ and SRS). Thus, neither categorical group status nor dimensional variation in these commonly implicated characteristics explained variability in neural vocal emotion processing. While subtle associations cannot be ruled out, these findings suggest that the preserved auditory socio-affective processing observed in the present study is unlikely to be masked by differences in alexithymia, depression, or quantitative autism characteristics.

### 4.5. Limitations and Future Directions

Although no evidence for reduced neural vocal emotion processing was observed, it is important to acknowledge that the present sample was not powered to detect very small between-group differences. Our sensitivity analysis indicated that only relatively large effects (Cohen’s *d* > 0.79) could be detected with 80% power. Consequently, subtle group differences may have remained undetected and the results should not be interpreted as evidence of complete equivalence between autistic and non-autistic adults.

A second limitation concerns the nature of the EEG paradigm itself. The current design captures implicit discrimination between emotional and neutral vocal utterances rather than the more complex emotion-processing demands encountered in everyday social interactions. Although frequency-tagging paradigms have repeatedly demonstrated high sensitivity in detecting subtle individual differences that may remain undetected using classical behavioral measures [41,42,43,44], they necessarily simplify the richness of real-world communication. Future studies could therefore increase ecological validity by directly contrasting multiple emotional categories within the same sequence or by introducing more dynamic and socially complex listening environments [76].

A third limitation concerns the composition of the sample. The study focused exclusively on intellectually able autistic adult men. This choice was motivated by both methodological and theoretical considerations. Autism is diagnosed more frequently in males than females [24], and gender differences have repeatedly been reported in emotion recognition and socio-affective processing [23,48,49]. Restricting the sample to men therefore reduced participant heterogeneity and maximized sensitivity for detecting autism-related differences in this initial application of the auditory frequency-tagging paradigm. However, this approach inevitably limits the generalizability of the findings. Autistic females may follow partly different developmental trajectories and may rely on distinct compensatory mechanisms during social perception. Future studies should therefore explicitly investigate whether the present findings extend to autistic women, individuals with intellectual disability, and other underrepresented subgroups across the autism spectrum.

### 4.6. Frequency-Tagging EEG as a Developmental Biomarker

Finally, the present findings underscore the importance of studying vocal emotion processing earlier in development. Difficulties in understanding emotional signals can contribute to social exclusion, peer rejection, and reduced social participation, particularly during childhood and adolescence. Longitudinal work is therefore needed to determine when potential differences in vocal emotion processing emerge and how they relate to later developmental outcomes.

Importantly, auditory frequency-tagging EEG offers considerable promise as a translational tool for developmental research. Its high signal-to-noise ratio, short acquisition time, minimal task demands, and robust individual-level responses make it particularly well suited for use in infants, children, and clinical populations. Future longitudinal studies could leverage this approach to characterize developmental trajectories of socio-affective processing, identify early risk markers for social-communication difficulties, and evaluate the impact of targeted interventions on neural mechanisms of social perception. The observation that this paradigm detects reliable individual-level responses in both typical and at-risk populations further highlights its potential as a candidate biomarker for tracking developmental change and individual differences in socio-communicative functioning.

## 5. Conclusions

The present study provides converging neural and behavioral evidence for intact vocal emotion processing in autistic adult men. Using a sensitive and objective frequency-tagging EEG paradigm, we demonstrate preserved automatic discrimination of vocal emotional expressions, alongside typical performance on explicit auditory emotion-recognition measures. These findings support the view that socio-affective processing differences reported in autistic children may attenuate across development and are not necessarily evident in adulthood. More broadly, the combination of short testing times, excellent signal-to-noise ratio, minimal task demands, and reliable individual-level responses highlights the potential of frequency-tagging EEG as a promising biomarker for future developmental and longitudinal studies of socio-communicative functioning.

## Figures and Tables

**Table 1:** Participant characteristics.

	ASD Group (*N* = 25)		NA Group (*N* = 25)		Statistics	
	*Mean*	*SD*	*Mean*	*SD*	*T*	*p*
Age	26.7	5.5	25.6	4.0	0.85	0.397
Total IQ	108	20	106	14	0.29	0.775
Verbal IQ	106	16	105	12	0.37	0.714
Performance IQ	110	22	107	17	0.51	0.609

**Table 2:** Behavioral scores on the questionnaires and the Multimodal Emotion recognition Test.

	ASD Group (*N* = 25)		NA Group (*N* = 25)		Statistics	
	*Mean*	*SD*	*Mean*	*SD*	*T*	*p*
SRS	74.4	24.7	35.1	14.2	6.92	<0.001 ***
AQ	130.9	17.1	105	13.3	5.98	<0.001 ***
TAS-20	55.5	9.3	45	8.7	4.15	<0.001 ***
BDI	13.2	8.2	7.2	5.2	3.09	0.003 **
MERT average	52.1	7.6	53.2	6.9	0.53	0.601
MERT Auditory	42.9	9.3	42.9	10.3	0.00	0.998
MERT Audio-Visual	61.9	11.1	61.8	8.9	0.02	0.982
MERT Video	56.8	8.9	58.6	7.9	0.76	0.451
MERT Picture	46.9	8.8	49.6	8.4	1.09	0.282

Note. SRS: Social Responsiveness Scale, AQ: Autism spectrum Quotient, TAS-20: Toronto Alexithymia Scale, BDI: Beck Depression Inventory, MERT: Multimodal Emotion recognition Test. ** *p* < 0.01, *** *p* < 0.001.

**Table 3:** Number of subjects yielding significant oddball responses at the individual level for each condition.

Condition	ASD Group (*N* = 25)	NA Group (*N* = 25)
Fear	23	25
Anger	21	19
Happy	18	20
Sad	20	21
Scrambled	4	10
Total	86/125	95/125

## Data Availability

The newly designed Emotional Voices and Identity Database (EVID), created for this study and described in the manuscript, will be made publicly available, as well as the analysis scripts. The EEG data of the study will be anonymized and will be available upon request.

## Supplementary Materials

The following supporting information can be downloaded at: https://www.mdpi.com/article/10.3390/brainsci16080876/s1. Figure S1. Base-rate responses indexing general voice processing. Scalp topographies showing the spatial distribution of summed baseline-subtracted amplitudes at the base stimulation frequency and its harmonics (4 and 8 Hz) for each condition. Warmer colors indicate stronger neural synchronization to the periodic vocal stimulation, whereas cooler colors indicate weaker responses. Topographical maps are displayed separately for the non-autistic (NA; upper row) and ASD (lower row) groups, illustrating the highly similar spatial distribution of voice-selective responses across groups. Base-rate activity was predominantly centered over the left parietal (LP), medial occipital (MO), and right temporal (RT) scalp regions that were selected as regions of interest (ROIs) for the statistical analyses. Figure S2. Neural responses across conditions as a function of spatial location (ROI). Error bars represent the standard error of the mean (SEM). Top panel: Base-rate synchronization. Summed baseline-corrected amplitudes at the base-rate harmonics (4 and 8 Hz) are shown for each condition separately, as well as for the average across emotional conditions. Bottom panel: Oddball discrimination. Summed baseline-corrected amplitudes at the oddball frequencies are shown for each condition separately, as well as for the average across emotional conditions. MO: Medial Occipital, LP: Left Parietal, RP: Right Parietal, LT: Left Temporal, RP: Right Temporal, MF: Medial Frontal.
