# Supplementary material for: Intact Neural and Behavioral Processing of Vocal Emotional Expressions in Men with Autism
Source: Brain Sci. 2026 Aug 18;16(8):876. doi: 10.3390/brainsci16080876 (PMC13510860; doi:10.3390/brainsci16080876)
Supplement: Supplementary file 1 [file brainsci-16-00876-s001.zip › brainsci-4440717-supplementary.pdf]

Supplementary figures

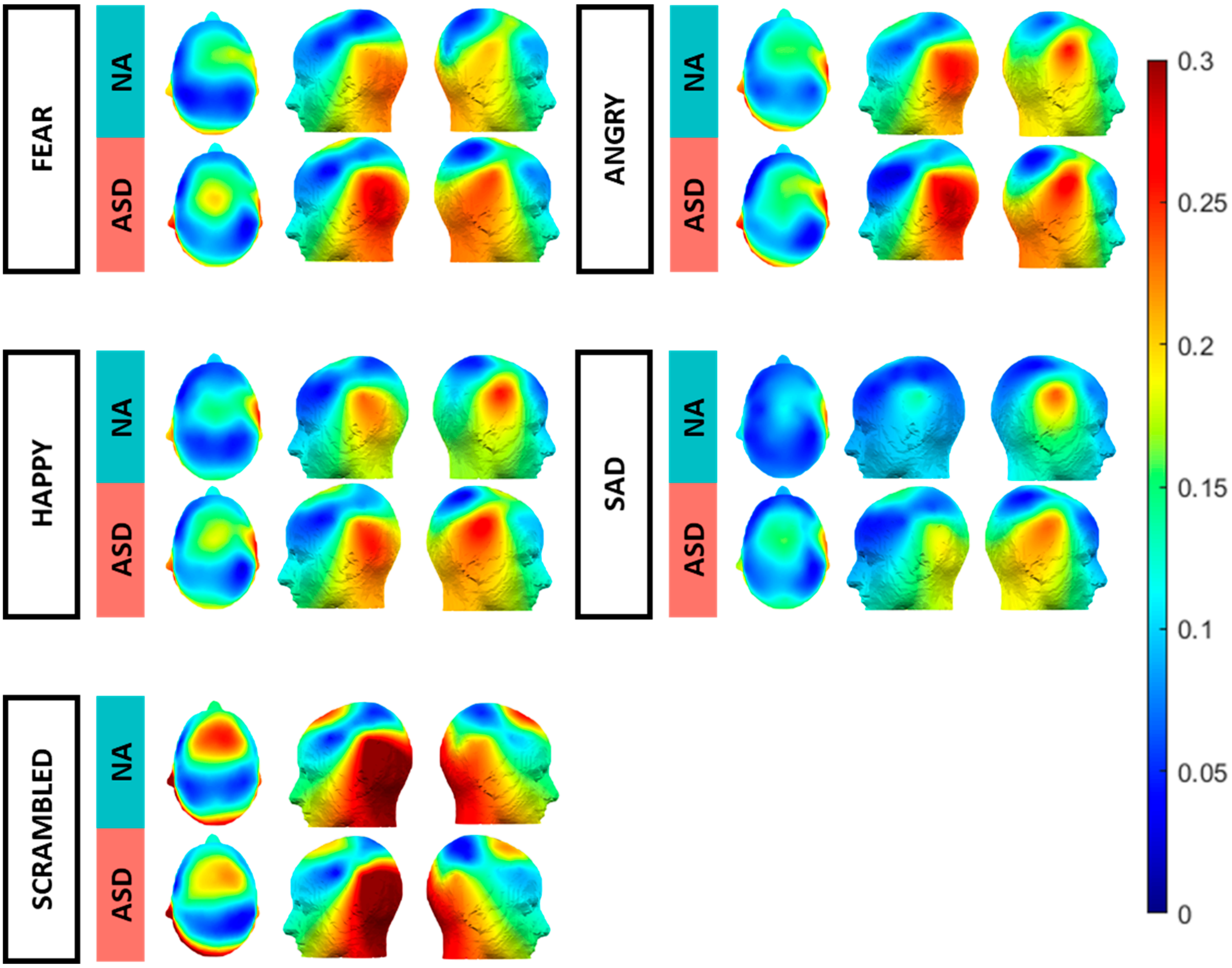

Figure S1. ***Base-rate responses indexing general voice processing.*** *Scalp topographies showing the spatial distribution of summed baseline-subtracted amplitudes at the base stimulation frequency and its harmonics (4 and 8 Hz) for each condition. Warmer colors indicate stronger neural synchronization to the periodic vocal stimulation, whereas cooler colors indicate weaker responses. Topographical maps are displayed separately for the non-autistic (NA; upper row) and ASD (lower row) groups, illustrating the highly similar spatial distribution of voice-selective responses across groups. Base-rate activity was predominantly centered over the left parietal (LP), medial occipital (MO), and right temporal (RT) scalp regions that were selected as regions of interest (ROIs) for the statistical analyses.*

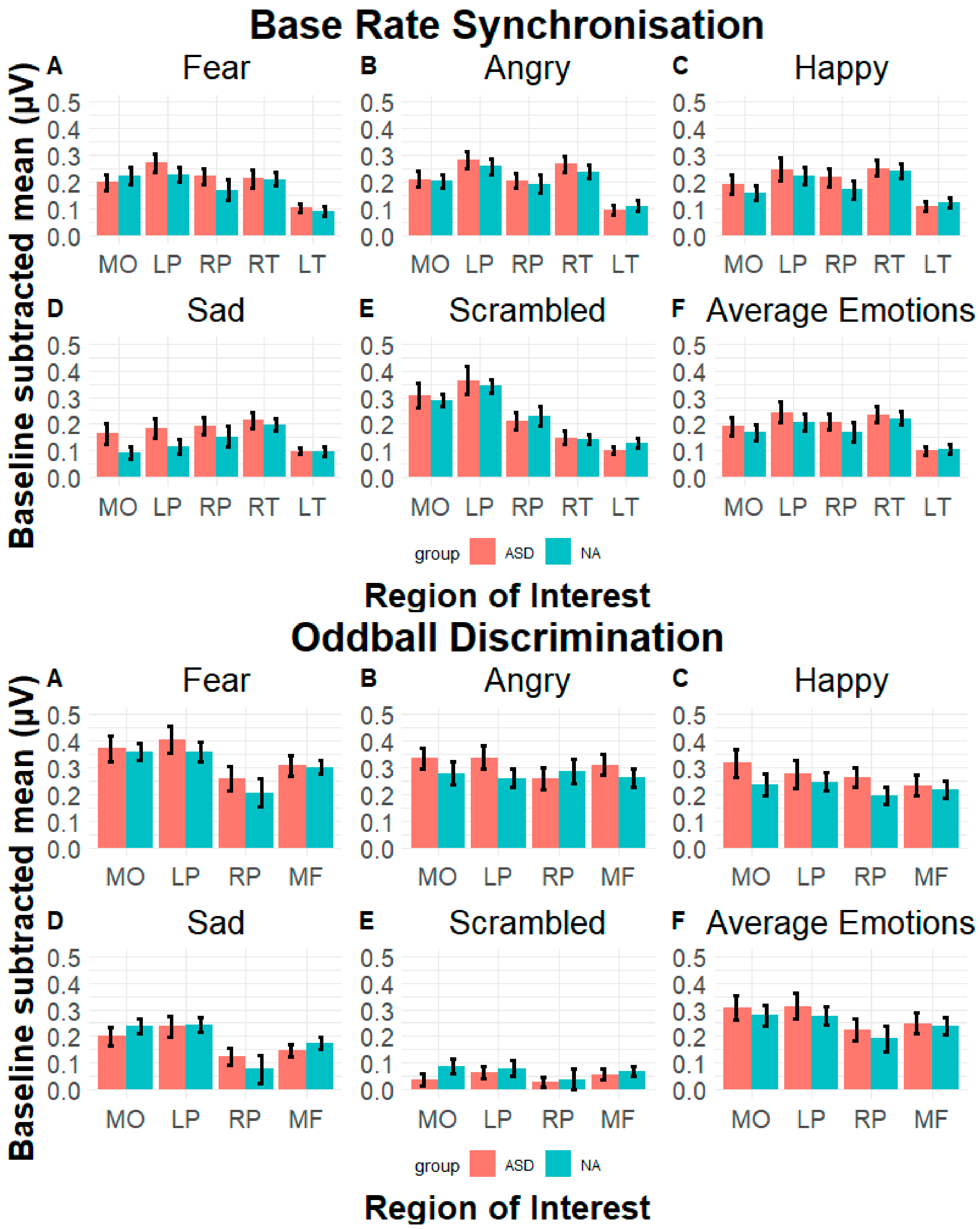

Figure S2. ***Neural responses across conditions as a function of spatial location (ROI).*** *Error bars represent the standard error of the mean (SEM).* ***Top panel****: Base-rate synchronization. Summed baseline-corrected amplitudes at the base-rate harmonics (4 and 8 Hz) are shown for each condition separately, as well as for the average across emotional conditions.* ***Bottom panel****: Oddball discrimination. Summed baseline-corrected amplitudes at the oddball frequencies are shown for each condition separately, as well as for the average across emotional conditions.MO: Medial Occipital, LP: Left Parietal, RP; Right Parietal, LT: Left Temporal, RP: Right Temporal, MF: Medial Frontal.*
